# T helper cell-related changes in peripheral blood induced by progressive effort among soccer players

**DOI:** 10.1371/journal.pone.0227993

**Published:** 2020-01-28

**Authors:** Dorota Kostrzewa-Nowak, Robert Nowak

**Affiliations:** Centre for Human Structural and Functional Research, University of Szczecin, Szczecin, Poland; Universidade Federal de Juiz de Fora, BRAZIL

## Abstract

**Objectives:**

The regulatory mechanisms affecting the modulation of the immune system accompanying the progressive effort to exhaustion, particularly associated with T cells, are not fully understood. We analysed the impact of two progressive effort protocols on T helper (Th) cell distribution and selected cytokines.

**Methods:**

Sixty-two male soccer players with a median age of 17 (16–29) years performed different protocols for progressive exercise until exhaustion: YO-YO (YYRL1) and Beep. Blood samples for all analyses were taken three times: at baseline, post-effort, and in recovery.

**Results:**

The percentage of Th1 cells increased post-effort and in recovery. The post-effort percentage of Th1 cells was higher in the Beep group compared to the YYRL1 group. Significant post-effort increase in Th17 cells was observed in both groups. The post-effort percentage of regulatory T cells (Treg) increased in the Beep group. An increased post-effort concentration of IL-2, IL-6, IL-8 and IFN-γ in both groups was observed. Post-effort TNF-α and IL-10 levels were higher than baseline in the YYRL1 group, while the post-effort IL-17A concentration was lower than baseline only in the Beep group. The recovery IL-2, IL-4, TNF-α and IFN-γ levels were higher than baseline in the YYRL1 group. The recovery IL-4, IL-6, IL-8, TNF-α and IFN-γ values were higher than baseline in the Beep group.

**Conclusion:**

The molecular patterns related to cytokine secretion are not the same between different protocols for progressive effort. It seems that Treg cells are probably the key cells responsible for silencing the inflammation and enhancing anti-inflammatory pathways.

## Introduction

Physical effort induces significant disorders of homeostasis on a physiological, immunological and molecular level [1–8]. Although the role of peripheral leukocytes is widely discussed in the literature [9–16], the regulatory mechanisms affecting the modulation of the immune system, especially T cells, which accompany the progressive effort to exhaustion are not fully understood.

It has been widely discussed that one of the characteristics of immune system aging is a change in T cell subsets, namely central memory, effector memory and aging T cells [17]. Simpson postulates that the total counts of lymphocytes usually reaches baseline values in the peripheral blood up to 24 hours after the effort [18]. It was also shown that the changes in the distribution of T cell subsets, T helper (Th) and T cytotoxic (Tc), following three days of high-intensity interval exercises results from the mobilization of proapoptotic proteins and migration of lymphocytes from lymphoid tissues to peripheral blood [19].

Changes in the distribution of Th1 and Th2 cell subsets as a consequence of the post-exercise cytokine secretion of participants (including runners and triathletes) and professional athletes (including marathoners and rowers) in different age groups favours the emergence of type 2 cell subsets (T2, including Th2 and Tc2) [20–24]. In addition, regulatory T cells (Treg) have recently been identified as the cells promoting the repair of muscle fibres through the secretion of autocrine growth factor amphiregulin in the muscle tissue [25]. The proportion of Th lymphocyte subsets, including Th1, Th2, Th17 or Treg, involved in the modulation of the immune response following physical activity is key to silencing or enhancing post-effort immune changes. Importantly, the participation of these cells leads not only to local immune changes, but may also underlie the post-effort modulation of the immune response at the systemic level. From this perspective, Th cell subsets appear to be the best candidates to understand biological mechanisms of adaptation to physical effort in professional athletes.

From a practical point of view, the molecular mechanisms behind the post-effort alterations are not as important as a better understanding of the impact of the endurance protocol test on the immune response on a physiological level. Therefore, it seems to be important to verify if different progressive test until exhaustion protocols commonly used in sports practice, e.g. YO-YO intermittent recovery test level 1 [26] and the maximal multistage 20 m shuttle run test [27, 28], induce the same cellular and signalling changes.

Taking this data into account, the aim of this study was to assess the impact of the endurance effort on Th cell subset distribution on a physiological level and the post-effort changes in cytokine levels related to Th cells on a functional level.

## Materials and methods

### Participants

Sixty-two male soccer players (excluding goalkeepers), median age 17 years old (range, 16–29 years), with at least 6 years of training experience were recruited for this study. The participants were divided into two groups performing different protocols of the progressive exercise until exhaustion, namely the YO-YO intermittent recovery test level 1 (YYRL1) protocol [26] and the maximal multistage 20 m shuttle run (Beep) [27, 28].

All participants qualified for the study belong to the same sports club and took part in the same annual macrocycle training program. The experiments were performed after two weeks of summer vacation, when the participants were asked to refrain from physical effort, especially training units.

Participants had no history of any metabolic syndrome (according to the International Diabetes Federation description) [29] or cardiovascular diseases (defined by WHO) [30]. They were non-smokers and refrained from taking any medications or supplements known to affect metabolism. Since the exercise protocols are routinely performed by the soccer club, recruitment of the participants consisted of informing them (and their parents, when appropriate) about the study and inviting them to take part in it by donating extra blood samples.

All athletes not meeting the inclusion criteria (e.g. not giving us or later withdrawing the consent to participate, goalkeepers) were excluded from the study.

### Progressive test protocols

Both exercise tests evaluate athlete’s aerobic capacity.

During YO-YO intermittent recovery test level 1 (YYRL1) athletes performed 2 x 20 m (back and forth) shuttle runs at increasing speed (starting at the speed of 10 km/h) where each shuttle is separated by 10 s (2 x 5 m; back and forth) of active recovery (a jogtrot). The timing was controlled by the audio signal. Athletes performed the test as long as they were unable to maintain the speed (not reaching the finish line before or at sounding the audio signal two times at a row).

Maximal multistage 20 m shuttle run (Beep) test is similar to the YYRL1 test. However, it lacks the stage of active recovery consisting only from 20 m shuttle runs (back and forth) between audio signals at increasing speed (starting at the speed of 8.5 km/h). Similarly to YYRL1 test, during the Beep test, athletes performed the test as long as they were unable to maintain the speed (not reaching the finish line before or at sounding the audio signal two times at a row).

## Methods

The study was approved by the Local Ethics Committee at the Regional Medical Chamber in Szczecin (no. 03/KB/VI/2017). Participants (and their parents, when appropriate) were fully informed of any risks and possible discomfort associated with the experimental procedures before giving their written consent to participate.

Body mass and body composition parameters (body mass index (BMI), basal metabolic rate (BMR), percentage of fat (FAT), fat free mass (FFM) and total body water (TBW)) of the participants were determined using a Body Composition Analyser Tanita BC-418MA (Tanita, Tokyo, Japan).

To analyse the impact of progressive effort on Th cell distribution and levels of selected cytokines, blood samples were taken three times: before the test to assess the baseline values (baseline), immediately after the progressive test until exhaustion (post-effort, no longer than 5 minutes after the test) to assess a short-time post-effort effects, and during recovery time (recovery, about 17 hours after the test) to assess long-term biological effects. Seventeen hours of recovery represents the mean longest period between two physical efforts, e.g. between a soccer match and the next training or between two trainings. At each time point, blood samples were collected in 7.5 mL S-Monovette tubes with ethylenediaminetetraacetic acid (EDTA K3, 1.6 mg EDTA/mL blood; SARSTEDT AG & Co., Nümbrecht, Germany). All analyses were performed immediately after blood collection.

White blood cell (WBC) and lymphocyte (LYM) counts were analysed using haematology analyser ABX Micros 60 (Horiba ABX, Warsaw, Poland).

Lymphocytes for future analyses of Th cell subsets were isolated from peripheral blood using density gradient separating medium (Corning, Manassas, VA, USA) and were frozen at -80 ^o^C using the standard protocol for in vitro cell line storage [31–34].

To determine percentages of Th1, Th2 and Th17 cell subsets the Human Th1/Th2/Th17 Phenotyping Kit (BD Biosciences, San Jose, CA, USA) was used. The antibodies cocktail contained: FITC-labelled interferon-gamma (clone B27), PE-labelled IL-17A (clone N49-653), PerCP-Cy5.5-labelled CD4 (clone SK3), and APC-labelled IL-4 (clone MP4-25D2). To measure Treg cell subsets, the Human Th17/Treg Phenotyping Kit (BD Pharmingen^™^, San Jose, CA, USA) were used, as described previously [20]. The antibodies cocktail contained: PE-labelled IL-17A, PerCP-Cy5.5-labelled CD4, and Alexa Fluor® 647-labelled FoxP3. The analyses were performed using the BD Accuri^™^ C6 flow cytometer (Becton Dickinson, Franklin Lakes, NJ, USA). For each sample, the fluorescence signal of at least 10^4^ events gated for the forward and side light-scatter characteristics of lymphocytes was measured. The results were calculated using BD Accuri^™^ C6 (ver. 1.0.264.21) and FCS Express (ver. 4.07.0020 RUO Edition; De Novo Software, Los Angeles, CA, USA) software.

The measurement of selected cytokines, namely interleukin-2 (IL-2), -4 (IL-4), -6 (IL-6), -8 (IL-8), -10 (IL-10), 12p70 (IL-12p70), -17A (IL-17A), tumour necrosis factor alpha (TNF-α) and interferon gamma (IFN-γ), was performed using BD Cytometric Bead Array (CBA) Human Inflammatory Cytokines Kit (BD Biosciences) and analysed using the BD Accuri^™^ C6 flow cytometer according to manufacturer’s protocol, as previously described [20, 35]. For each sample, the fluorescence signal of 2100 events gated for capture beads population was measured. Results were calculated using FCAP Array^™^ Software (ver. 3.0.1; Soft Flow Hungary Ltd., Pecs, Hungary).

### Statistical analysis

All data are presented as median (Q1-Q3), except for age, which is presented as median (min-max). Statistical analysis was performed using STATISTICA (data analysis software system), version 13 software (TIBCO Software Inc., 2017). Significant differences between analysed time points (baseline vs. post-effort vs. recovery) were calculated using Friedman's analysis of variance for repeated measures followed by post-hoc Dunn’s test with Bonferroni correction. Significant differences between YYRL1 and Beep groups were calculated using the Mann-Whitney U test. For each analysis, a p-value < 0.05 was considered to be significant.

## Results

### Participants’ characteristics

The baseline characteristics of the participants qualified for each study groups are presented in Table 1. Statistical analysis conformed homogeneity of the groups at baseline.

**Table 1:** Baseline characteristics of the participants and the cardiorespiratory fitness measures of participants during the progressive test until exhaustion.

	YYRL1 group (n = 31)	Beep group (n = 31)	*P*_*M-W*_
Age [years]	17 (16–29)	17 (16–22)	0.385
Height [cm]	176 (169–184)	179 (173–183)	0.585
Weight [kg]	66.9 (63.1–71.9)	71.0 (64.5–73.5)	0.125
FAT [%]	9.4 (6.7–12.2)	9.8 (5.9–12.6)	0.867
FAT MASS [kg]	6.5 (4.8–8.0)	6.9 (4.2–8.2)	0.780
FFM [kg]	59.7 (56.6–67.1)	64.5 (58.8–68.0)	0.133
TBW [kg]	43.7 (41.4–49.1)	47.2 (43.0–49.8)	0.125
Length of training experience [years]	10.0 (9.0–12.0)	11.0 (8.0–12.0)	0.780
Weekly training volume [hours]	12.0 (10.0–15.0)	12.0 (10.0–12.0)	0.202

The table presents median (Q1-Q3) values (except for the age, where median (min-max) is presented) characterising the participants.

Significance levels of differences observed between analysed groups (YYRL1 vs. Beep) were assessed using Mann-Whitney U test (*p*_*M-W*_—Mann-Whitney p values).

n–number of participants; Beep—maximal multistage 20m shuttle run test; YYRL1—YO-YO intermittent recovery test level 1 protocol; FAT–percentage of fat; FFM–fat free mass; TBW–total body water.

The analysis of WBC and LYM total counts in peripheral blood indicated that the progressive effort induced a significant increase in both parameters after the tests in comparison to baseline as well as recovery values (Table 2). There were no significant differences between YYRL1 and Beep group at each time point.

**Table 2:** White blood cell (WBC) and lymphocyte (LYM) counts of study participants blood samples.

Variable	YYRL1 group (n = 31)				Beep group (n = 31)			
	baseline	post-effort	recovery	*p*_*F*_	baseline	post-effort	recovery	*p*_*F*_
WBC [10^9^/L]	5.5^aa^ (4.5–6.4)	8.1^bbb^ (6.2–9.5)	5.5 (4.5–6.0)	*< 0*.*001*	5.3^aaa^ (4.5–5.7)	8.1^bbb^ (6.8–9.9)	5.1 (3.9–6.1)	*< 0*.*001*
LYM [10^9^/L]	1.9^aaa^ (1.5–2.3)	2.9^bbb^ (2.3–3.9)	1.7 (1.5–2.1)	*< 0*.*001*	1.8^aaa^ (1.5–2.1)	3.5^bbb^ (2.7–4.2)	1.9 (1.8–2.3)	*< 0*.*001*

The table presents median (Q1-Q3) values.

The analyses were performed before (baseline) and after the progressive effort (5 minutes post-effort), and during the recovery time (about 17 hours after the test).

Significance levels of differences observed between analysed time points (pre-exercise vs. post-exercise vs. recovery) were assessed using Friedman's analysis of variance for repeated measures (*p*_*F*_—Friedman’s ANOVA p values) followed by post-hoc Dunn’s test with Bonferroni correction.

Post-hoc p values

^aa^ p < 0.01 or ^aaa^ p < 0.001 for baseline vs. post-effort

^bbb^ p < 0.001 for post-effort vs. recovery.

n–number of participants; Beep–maximal multistage 20 m shuttle run test; YYRL1 –YO-YO intermittent recovery test level 1 protocol.

### Th lymphocyte analysis

The percentage of Th1 cells in the YYRL1 and Beep groups was significantly higher after the test and at the recovery time point in comparison to baseline values (Table 3). The post-effort percentage of the Th1 cell subset was about 1.3-fold higher in the Beep group compared to the YYRL1 group (p < 0.001), while there were no statistically significant differences between baseline values in either study group. The progressive effort induced by different protocols did not influence the Th2 cell percentage (Table 3). Significant post-effort increases in percentages of Th17 cells were observed in both groups, but only in the YYRL1 group this value was increased at each studied time point compared to baseline (Table 3). Interestingly, there were no significant changes in post-effort Treg cell percentage in comparison to baseline found in the YYRL1 group, but the recovery value was about 1.4-fold lower than the values observed in the baseline and post-effort time points. On the other hand, the Treg cell percentage in the Beep group increased at each time point and was significantly higher than the baseline values 17 hours after the progressive effort (Table 3).

**Table 3:** Th cell subsets in isolated lymphocytes from peripheral blood of study participants.

Variable	YYRL1 group (n = 31)				Beep group (n = 31)			
	baseline	post-effort	recovery	*p*_*F*_	baseline	post-effort	recovery	*p*_*F*_
Th1 [%]	21.7^a^ (20.5–22.4)	23.6 (21.5–25.7)	25.0^ccc^ (23.5–26.2)	*< 0*.*001*	20.8^aaa^ (19.0–24.1)	31.8^b^ (28.8–35.1)	25.8^cc^ (24.0–31.5)	*< 0*.*001*
Th2 [%]	10.1 (9.1–10.7)	10.3 (9.8–11.2)	11.1 (9.9–11.3)	*0*.*160*	10.2 (8.6–11.0)	10.3 (9.2–12.7)	10.1 (10.0–11.1)	*0*.*471*
Th17 [%]	15.8^aaa^ (14.0–16.5)	22.4^bbb^ (19.2–23.5)	30.2^ccc^ (29.4–32.8)	*< 0*.*001*	10.0^aa^ (8.0–19.3)	17.9 (12.1–23.5)	22.8^ccc^ (18.5–24.4)	*< 0*.*001*
Treg [%]	6.2 (4.8–9.1)	6.8^bbb^ (5.7–8.3)	4.5^cc^ (3.3–6.5)	*< 0*.*001*	7.6^aaa^ (5.9–9.0)	13.3 (9.4–14.9)	12.7^ccc^ (10.2–13.8)	*< 0*.*001*

The table presents median (Q1-Q3) values.

The analyses were performed before (baseline) and after the progressive effort (5 minutes post-effort) and during recovery time (about 17 hours after the test).

Significance levels of differences observed between analysed time points (pre-exercise vs. post-exercise vs. recovery) were assessed using Friedman's analysis of variance for repeated measures (*p*_*F*_—Friedman’s ANOVA p values) followed by post-hoc Dunn’s test with Bonferroni correction.

Post-hoc p values

^a^ p < 0.05

^aa^ p < 0.01 or ^aaa^ p < 0.001 for baseline vs. post-effort

^b^ p < 0.05 or ^bbb^ p < 0.001 for post-effort vs. recovery

^cc^ p < 0.01 or ^ccc^ p < 0.001 for baseline vs. recovery

n–number of participants, Beep–maximal multistage 20 m shuttle run test, YYRL1 –YO-YO intermittent recovery test level 1 protocol.

### Plasma cytokine analysis

The cytokine profile in plasma is presented in Table 4. The progressive effort protocols induced a significantly increased post-effort concentration of IL-2, IL-6, IL-8 and IFN-γ in both study groups compared to the baseline values. Post-effort levels of TNF-α and IL-10 were significantly higher than the baseline value in the YYRL1 group, while the post-effort concertation of plasma IL-17A was lower than the baseline concentration only in the Beep group. The recovery levels of IL-2, IL-4, TNF-α and IFN-γ were higher than baseline in the YYRL1 group. Seventeen hours after the progressive effort protocols were performed the values of IL-4, IL-6, IL-8, TNF-α and IFN-γ were higher than the baseline values in the Beep group. No significant changes in IL-12p70 were found in either study group.

**Table 4:** Median level of interleukin-2 (IL-2), interleukin-4 (IL-4), interleukin-6 (IL-6), interleukin-8 (IL-8), interleukin-10 (IL-10), interleukin-12p70 (IL-12p70), interleukin-17A (IL-17A), tumour necrosis factor alfa (TNF-α) and interferon gamma (IFN-γ) of study participant’s plasma samples.

Variable	YYRL1 group (n = 31)				Beep group (n = 31)			
	baseline	post-effort	recovery	*p*_*F*_	baseline	post-effort	recovery	*p*_*F*_
IL-2 [pg/mL]	2.36^aaa^ (1.47–2.78)	11.29 (7.05–13.60)	14.56^ccc^ (12.58–16.29)	*< 0*.*001*	1.16^aaa^ (0.68–1.35)	3.26^bbb^ (2.36–9.80)	1.31 (0.99–2.04)	*< 0*.*001*
IL-4 [pg/mL]	1.26 (0.87–3.25)	2.25 (1.27–3.95)	2.58^c^ (1.29–3.95)	*0*.*024*	1.81^aaa^ (1.46–2.36)	13.33^b^ (9.86–18.70)	7.65^ccc^ (4.35–9.28)	*< 0*.*001*
IL-6 [pg/mL]	2.05^a^ (1.06–2.62)	4.33 (1.46–5.88)	3.03 (1.36–4.57)	*0*.*014*	1.65^a^ (1.30–2.00)	2.37 (1.42–3.69)	2.36^cc^ (1.69–3.46)	*0*.*003*
IL-8 [pg/mL]	2.41^aaa^(1.57–4.15)	14.45^bbb^ (12.36–17.40)	5.65 (3.65–8.26)	*< 0*.*001*	2.40^aaa^ (2.00–3.89)	6.49 (4.63–8.22)	8.97^ccc^ (3.57–14.14)	*< 0*.*001*
IL-10 [pg/mL]	1.05^a^ (0.60–1.36)	2.36 (1.25–2.65)	1.32 (0.79–2.13)	*0*.*018*	1.86 (1.51–2.15)	2.11 (1.53–2.41)	2.86 (1.77–5.59)	*0*.*068*
IL-12p70 [pg/mL]	1.60 (1.24–2.32)	1.29 (0.79–1.99)	1.46 (1.25–2.48)	*0*.*908*	0.99 (0.53–1.24)	1.24 (0.80–1.36)	2.31 (0.66–4.51)	*< 0*.*001*
IL-17A [pg/mL]	0.99 (0.36–1.55)	2.67 (1.85–3.80)	1.27^ccc^ (0.41–4.56)	*0*.*001*	2.66^aa^ (1.86–5.49)	1.36 (0.92–1.79)	1.25^ccc^ (0.62–1.88)	*< 0*.*001*
TNF-α [pg/mL]	1.29^aaa^ (0.88–2.46)	2.35 (1.97–3.21)	2.66^ccc^ (2.16–3.65)	*< 0*.*001*	0.90 (0.62–1.24)	1.30 (0.99–1.61)	1.87^ccc^ (1.05–3.50)	*< 0*.*001*
IFN-γ [pg/mL]	2.35^aaa^ (1.29–2.58)	12.36 (10.08–14.55)	14.50^ccc^ (12.58–17.20)	*< 0*.*001*	1.86^aaa^ (1.26–2.35)	5.73 (4.99–8.83)	8.20^ccc^ (5.84–11.27)	*< 0*.*001*

The table presents median (Q1-Q3) values.

The analyses were performed before (baseline) and after the progressive effort (5 minutes post-effort) and during the recovery time (about 17 hours after the test).

Significant differences observed between analysed time points (pre-exercise vs. post-exercise vs. recovery) were assessed using Friedman's analysis of variance for repeated measures (*p*_*F*_—Friedman’s ANOVA p values) followed by post-hoc Dunn’s test with Bonferroni correction.

Post-hoc p values

^a^ p < 0.05

^aa^ p < 0.01, or ^aaa^ p < 0.001 for baseline vs. post-effort

^b^ p < 0.05 or ^bbb^ p < 0.001 for post-effort vs. recovery

^c^ p < 0.05, ^cc^ p < 0.01 or ^ccc^ p < 0.001 for baseline vs. recovery.

n–number of participants; Beep—maximal multistage 20 m shuttle run test; YYRL1—YO-YO intermittent recovery test level 1 protocol.

## Discussion

### Th cell subset alterations

Post-effort leucocytosis and lymphocytosis is a well-known phenomenon in laboratory diagnostics and sport medicine. There is a great deal of data demonstrating that it is a short-term disorder and the physiological explanation of this observation is post-effort dehydration [36–41]. Haematological analysis performed up to 24 hours after intensive effort showed the presence of young subsets of leucocytes in peripheral blood [17, 18, 35]. Taking the pleiotropic role of different leucocyte subsets in formation of immunity into account, it seems that their participation in the activation of the post-effort immune response pathways is as significant as it is in the case of response to antigenic stimuli. Essentially, intense exercise of short duration is associated with much weaker stimulation of the immune system than the stimulation associated with long-term effort of high intensity [42, 43]. Moreover, exercise of high and very high intensity increases the risk of upper respiratory tract infections in contrast to regular moderate intensity physical activity [36, 42, 44, 45]. Our previous study showed that Th cells play an important role in regulation and modulation of the immune response to progressive effort until exhaustion performed on a mechanical treadmill [20, 35]. The present study confirms that the immune response induced by the progressive effort is associated with the Th1 but not Th2 cell subset. Brown et al. suggested that the changes in distribution of Th lymphocytes in the peripheral blood as a result of increasing training loads indicates that they are less important in modulation of post-exercise immune response [46].

Interestingly, the findings from our research show a significant increase in the Th17 cell subset during the recovery after the YO-YO intermittent recovery test level 1 protocol and maximal multistage 20 m shuttle run test. There were no significant changes in the percentage of Th17 cells among soccer players after the progressive effort performed on a mechanical treadmill [20]. This disagreement may be explained by the difference in protocol for the progressive effort. Both YYIR1 and Beep tests consists of intermittent running back and forth, thus requiring from the athlete to stop to change the direction of the run, whereas the test on mechanical treadmill is continuous one and, more importantly, experienced athletes can use the treadmill’s drive to “carry” them slightly reducing the effort. Moreover, it was found in this study that the progressive effort induced a decrease in Treg cells during the recovery after the YO-YO test in contrast to the Beep test as well as the test performed on a mechanical treadmill [20]. The Treg cells express the FoxP3 transcription factor and are critical for the prevention of excess immunopathology or autoimmunity through multiple mechanisms [25]. The increase in Th17 cells suggests a more rapid immunological response induced by the progressive effort and indicates that the biological mechanisms of recovery are related to different immunological pathways depending on the test protocol. This hypothesis seems to be confirmed by the different distribution of Treg cells 17 hours after the test. The Treg cell increase was observed only in the Beep group in our study. It is possible that, a post-effort inflammatory mechanism promoted by Th17 cells is silenced by Treg cells. These observations are in line with our previous study [20].

### Post-effort cytokine release

Similarly to our study, Kakanis et al. observed post-effort secretion of both Th1 and Th2 cell-related cytokines (IL-2 and TNF-α, or IL-6 and IL-10, respectively) by T cells stimulated with phytohemagglutinin [47]. The cytokine profile is not similar on the functional level in either group of soccer players studied. Our study demonstrated that the progressive effort induced an increase in IL-2, IL-4, IL-6, IL-8 and IFN-γ concentrations regardless of the test protocol used. Additionally, post-effort increases in IL-10 and TNF-α levels were only seen in the YYRL1 group. According to literature data, the changes in pro- and anti-inflammatory cytokine levels depend on the intensity and duration of physical effort [48–50]. The increase in IL-2 concentration observed in our study is in accordance with the increase in the Th1 cell subset distribution. IL-2 is involved in the proliferation and activation of T cells [51–53], including differentiation of naïve T cells [54, 55], which may explain the increase in Th cell percentage at the post-effort and recovery time points. Interestingly, the level of this cytokine increased during the recovery time only in the YYRL1 group, probably because the Th1-related pathways involved in post-effort response in these athletes lasts longer than in the soccer players assigned to the Beep group. On the other hand, an increase in IL-4 levels observed only in the Beep group suggests more rapid pro-inflammatory response even though there were no significant increases in the percentage of Th2 cells. As was found in our previous study, NK cells are the most intensively recruited immediately after the endurance effort [35]. The role of post-effort responses related to NK cells may explain this phenomenon.

It was shown that high-intensity exercise leads to an increase in TNF-α, IL-1, IL-6 and IL-1 receptor antagonist in plasma, and an increase in the expression of TNF-α, IL-10, IL-8 receptors and inflammatory protein macrophage-1 in highly qualified athletes [48, 49]. IL-6 is responsible for co-activation of T cells, is associated with their proliferation and does not inhibit IL-2 production [56], which may explain the higher concentrations of those interleukins found in our study even though there were no significant changes in the percentage of the Th2 cells, which are related to a more rapid pro-inflammatory immune response. It was also found that the endurance effort tests on the cycle ergometers and the mechanical treadmill do not have volume or intensity enough to trigger muscle cells to stimulate the secretion of pro-inflammatory cytokines including IL-6 [57–59]. Our previous study showed that the IL-6 levels were significantly increased 17 hours after the progressive effort on a mechanical treadmill [20, 35], while in the present study it was increased immediately after the completion of the Beep and YO-YO tests. It is worth noting that in contrast to the present study, progressive effort on the mechanical treadmill did not trigger changes in Th17 cell percentage. The increase in IL-6 plasma level is one of the most probable promoters of Th17 cell differentiation [12, 60]. This observation helps to explain our findings of a post-effort increase in Th17 cell percentage without an increase in IL-17A levels.

Numerous reports have shown that long-term endurance efforts [48, 49, 61], as well as moderate intensity exercises [62, 63] and progressive effort on the mechanical treadmill [20, 35] caused an increase in IL-8 levels. This is in line with our findings. IL-8 is known to have the pleiotropic and haematopoietic role and also belongs to the family of factors related to angiogenesis, proliferation, invasion and migration of cells. It is described as a chemotactic factor for neutrophils [64–67]. Taking all that into account, it seems that this cytokine is also a key factor in post-effort immune signalling pathways.

On the other hand, it has been widely acknowledged that in the post-effort anti-inflammatory response, IL-10 appears in the circulation [68–73]. It is also an important molecule related to the regeneration of muscle tissue [69]. The lack of significant change in IL-10 in the Beep group seen in this study is not in line with our previous observations [20, 35] and suggests that outdoor running on an athletic track causes more pro-inflammatory response than that seen with progressive effort on mechanical treadmill.

No changes in IL-12p70 were found after the progressive effort, which may be related to the fact that the increase in IL-12 is predominantly associated with the maximum anaerobic effort [74, 75].

TNF-α is known to be involved in the inflammatory response to muscle damage [76]. This may offer a probable explanation of differences found between the results found after the YO-YO and Beep test. The TNF-α and IFN-γ profile found in both study groups is in line with the significant changes found in the distribution of Th cell subsets.

## Conclusions

The molecular patterns related to cytokine secretion are not the same for different protocols for progressive effort. On the other hand, the immune response induced by endurance progressive effort causes an increase in Th1 cells, while only the effort on athletic tracks induces an increase in Th17 cells. Interestingly, the progressive effort induces both Th1- (IL-2, TNF-α, IFN-γ) and Th2-related (IL-4, IL-6) cytokine release. From this point of view, it seems that Treg cells are probably the key cells responsible for the silencing the inflammation and enhancing the anti-inflammatory pathways.

In terms of immunomodulatory changes, our results help facilitate the choice of endurance test for diagnostic purposes depending on the training stage. It is particularly important during the competition season when it is necessary to carry out diagnostic tests to quickly correct/modify the training programme without additional burden on the player's immune system, especially in team sports where the games are played for about 38–36 weeks a year.

## Supporting information

S1 Table Raw data obtained during the study. (XLSX)

## References

[1] Akseki D, Akkaya G, Erduran M, Pinar H. Proprioception of the knee joint in patellofemoral pain syndrome. Acta Orthop Traumatol Turc. 2008;42(5): 316–321. 10.3944/aott.2008.316 19158451

[2] Arguis MJ, Perez J, Martínez G, Ubre M, Gomar C. Contralateral neuropathic pain following a surgical model of unilateral nerve injury in rats. Reg Anesth Pain Med 2008;33(3): 211–216. 10.1016/j.rapm.2007.12.003 18433671

[3] Banfi G, Colombini A, Lombardi G, Lubkowska A. Metabolic markers in sports medicine. Adv Clin Chem. 2012;56: 1–54. 10.1016/b978-0-12-394317-0.00015-7 22397027

[4] Fallon KE. The clinical utility of screening of biochemical parameters in elite athletes: analysis of 100 cases. Br J Sports Med 2008;42(5): 334–337. 10.1136/bjsm.2007.041137 18070805

[5] Gravina L, Ruiz F, Lekue JA, Irazusta J, Gil SM. Metabolic impact of a soccer match on female players. J Sports Sci 2011;29(12): 1345–1352. 10.1080/02640414.2011.597420 21777165

[6] Cadefau J, Casademont J, Grau JM, Fernández J, Balaguer A, Vernet M, et al Biochemical and histochemical adaptation to sprint training in young athletes. Acta Physiol Scand 1990;140(3): 341–351. 10.1111/j.1748-1716.1990.tb09008.x 2082703

[7] Meyer T, Meister S. Routine blood parameters in elite soccer players. Int J Sports Med 2011;32(11): 875–881. 10.1055/s-0031-1280776 22020850

[8] Wiacek M, Andrzejewski M, Chmura J, Zubrzycki IZ. The changes of the specific physiological parameters in response to 12-week individualized training of young soccer players. J Strength Cond Res 2011;25(6): 1514–1521. 10.1519/JSC.0b013e3181ddf860 21386728

[9] Ahlers JD, Belyakov IM. Memories that last forever: strategies for optimizing vaccine T-cell memory. Blood 2010;115(9): 1678–1689. 10.1182/blood-2009-06-227546 19903895 PMC2920202

[10] de Abreu MS, Giacomini ACVV, Zanandrea R, Dos Santos BE, Genario R, de Oliveira GG, et al Psychoneuroimmunology and immunopsychiatry of zebrafish. Psychoneuroendocrinology 2018;92: 1–12. 10.1016/j.psyneuen.2018.03.014 29609110

[11] Hyldahl RD, Hubal MJ. Lengthening our perspective: morphological, cellular, and molecular responses to eccentric exercise. Muscle Nerve 2014;49(2): 155–170. 10.1002/mus.24077 24030935

[12] Naufel AO, Aguiar MCF, Madeira FM, Abreu LG. Treg and Th17 cells in inflammatory periapical disease: a systematic review. Braz Oral Res 2017;31:e103 10.1590/1807-3107bor-2017.vol31.0103 29267664

[13] Peake JM, Neubauer O, Walsh NP, Simpson RJ. Recovery of the immune system after exercise. J Appl Physiol (1985) 2017;122(5): 1077–1087. 10.1152/japplphysiol.00622.2016 27909225

[14] Peake JM, Neubauer O, Della Gatta PA, Nosaka K. Muscle damage and inflammation during recovery from exercise. J Appl Physiol (1985) 2017;122(3): 559–570. 10.1152/japplphysiol.00971.2016 28035017

[15] Phadnis-Moghe AS, Kaminski NE. Immunotoxicity testing using human primary leukocytes: An adjunct approach for the evaluation of human risk. Curr Opin Toxicol 2017;3: 25–29. 10.1016/j.cotox.2017.04.005 29619412 PMC5880284

[16] Windsor MT, Bailey TG, Perissiou M, Meital L, Golledge J, Russell FD, et al Cytokine Responses to Acute Exercise in Healthy Older Adults: The Effect of Cardiorespiratory Fitness. Front Physiol 2018;9:203 10.3389/fphys.2018.00203 29599722 PMC5862854

[17] Spielmann G, McFarlin BK, O’Connor DP, Smith PJ, Pircher H, Simpson RJ. Aerobic fitness is associated with lower proportions of senescent blood T-cells in man. Brain Behav Immun 2011;25(8): 1521–1529. 10.1016/j.bbi.2011.07.226 21784146

[18] Simpson RJ. Aging, persistent viral infections, and immunosenescence: can exercise “make space”? Exerc Sport Sci Rev 2011;39(1): 23–33. 10.1097/JES.0b013e318201f39d 21088603

[19] Navalta JW, Tibana RA, Fedor EA, Vieira A, Prestes J. Three consecutive days of interval runs to exhaustion affects lymphocyte subset apoptosis and migration. Biomed Res Int 2014; 2014:694801 10.1155/2014/694801 24895602 PMC4033355

[20] Kostrzewa-Nowak D, Nowak R. Analysis of selected T cell subsets in peripheral blood after exhaustive effort among elite soccer players. Biochem Med (Zagreb) 2018;28(3):030707 10.11613/BM.2018.030707 30429675 PMC6214701

[21] Nowak R, Buryta R, Krupecki K, Zając T, Zawartka M, Proia P, et al The Impact of the Progressive Efficiency Test on a Rowing Ergometer on White Blood Cells Distribution and Clinical Chemistry Changes in Paralympic Rowers During the Preparatory Stage Before the Paralympic Games in Rio, 2016—A Case Report. J Hum Kinet 2017;60: 255–263. 10.1515/hukin-2017-0141 29340006 PMC5765806

[22] Ogawa K, Oka J, Yamakawa J, Higuchi M. Habitual exercise did not affect the balance of type 1 and type 2 cytokines in elderly people. Mech Ageing Dev 2003;124(8–9): 951–956. 10.1016/s0047-6374(03)00167-2 14499500

[23] Lancaster GI, Khan Q, Drysdale PT, Wallace F, Jeukendrup AE, Drayson MT, et al Effect of prolonged exercise and carbohydrate ingestion on type 1 and type 2 T lymphocyte distribution and intracellular cytokine production in humans. J Appl Physiol (1985) 2005;98: 565–571. 15322070 10.1152/japplphysiol.00754.2004

[24] Zhao G, Zhou S, Davie A, Su Q. Effects of moderate and high intensity exercise on T1/T2 balance. Exerc Immunol Rev 2012;18: 98–114. 22876723

[25] Zhang C, Li L, Feng K, Fan D, Xue W, Lu J. ‘Repair’ Treg Cells in Tissue Injury. Cell Physiol Biochem 2017;43(6): 2155–2169. 10.1159/000484295 29069643

[26] Bangsbo J. Fitness Training in Soccer: A Scientific Approach. Reedswain. Inc 2003.

[27] Léger LA, Lambert J. A maximal multistage 20-m shuttle run test to predict VO2 max. Eur J Appl Physiol Occup Physiol 1982;49(1): 1–12. 10.1007/bf00428958 7201922

[28] Metsios GS, Flouris AD, Koutedakis Y, Nevill A. Criterion-related validity and test-retest reliability of the 20m square shuttle test. J Sci Med Sport 2008;11(2): 214–217. 10.1016/j.jsams.2006.12.120 17544842

[29] IDF Consensus Worldwide Definition of the Metabolic Syndrome. Available from: https://www.idf.org/e-library/consensus-statements/60-idfconsensus-worldwide-definitionof-the-metabolic-syndrome (accessed 26 Jul 2019).

[30] WHO. Cardiovascular diseases. Available from: https://www.who.int/health-topics/cardiovascular-diseases/ (accessed 26 Jul 2019).

[31] Crowley LC, Scott AP, Marfell BJ, Boughaba JA, Chojnowski G, Waterhouse NJ. Measuring cell death by propidium iodide uptake and flow cytometry. Cold Spring Harb Protoc 2016;2016(7). 10.1101/pdb.prot087163 27371595

[32] Higdon LE, Lee K, Tang Q, Maltzman JS. Virtual global transplant laboratory standard operating procedures for blood collection, PBMC isolation, and storage. Transplant Direct 2016;2(9):e101 10.1097/TXD.0000000000000613 27795993 PMC5068195

[33] Lauruschkat CD, Wurster S, Page L, et al Susceptibility of A. fumigatus specific T-cell assays to pre-analytic blood storage and PBMC cryopreservation greatly depends on readout platform and analytes. Mycoses 2018;61(8): 549–560. 10.1111/myc.12780 29611226

[34] Yang J, Diaz N, Adelsberger J, Zhou X, Stevens R, Rupert A, et al The effects of storage temperature on PBMC gene expression. BMC Immunol 2016;17:6 10.1186/s12865-016-0144-1 26979060 PMC4791795

[35] Kostrzewa-Nowak D, Buryta R, Nowak R. Comparison of Selected CD45+ Cell Subsets' Response and Cytokine Levels on Exhaustive Effort Among Soccer Players. J Med Biochem 2019;38(3): 256–267. 10.2478/jomb-2018-0029 31156335 PMC6534948

[36] Keaney LC, Kilding AE, Merien F, Dulson DK. The impact of sport related stressors on immunity and illness risk in team-sport athletes. J Sci Med Sport 2018;21(12): 1192–1199. 10.1016/j.jsams.2018.05.014 29934212

[37] Neubauer O, Sabapathy S, Lazarus R, Jowett JB, Desbrow B, Peake JM, et al Transcriptome analysis of neutrophils after endurance exercise reveals novel signaling mechanisms in the immune response to physiological stress. J Appl Physiol (1985) 2013;114(12): 1677–1688. 10.1152/japplphysiol.00143.2013 23580600

[38] Peake J, Wilson G, Hordern M, Suzuki K, Yamaya K, Nosaka K, et al Changes in neutrophil surface receptor expression, degranulation, and respiratory burst activity after moderate- and high-intensity exercise. J Appl Physiol (1985) 2004;97(2): 612–861. 10.1152/japplphysiol.01331.2003 15075305

[39] Suzuki K, Totsuka M, Nakaji S, Yamada M, Kudoh S, Liu Q, et al Endurance exercise causes interaction among stress hormones, cytokines, neutrophil dynamics, and muscle damage. J Appl Physiol (1985) 1999;87(4): 1360–1367. 10.1152/jappl.1999.87.4.1360 10517764

[40] Walsh NP, Gleeson M, Shephard RJ, Gleeson M, Woods JA, Bishop NC, et al Position statement. Part one: Immune function and exercise. Exerc Immunol Rev 2011;17: 6–63. 21446352

[41] Wasinski F, Gregnani MF, Ornellas FH, Bacurau AV, Câmara NO, Araujo RC, et al Lymphocyte glucose and glutamine metabolism as targets of the anti-inflammatory and immunomodulatory effects of exercise. Mediators Inflamm 2014;2014:326803 10.1155/2014/326803 24987195 PMC4060061

[42] Nieman DC. Exercise immunology: practical applications. Int J Sports Med 1997;18 Suppl 1: S91–S100. 10.1055/s-2007-972705 9129268

[43] Gabriel H, Kindermann W. The acute immune response to exercise: what does it mean? Int J Sports Med 1997;18 Suppl 1:S28–S45. 10.1055/s-2007-972698 9129261

[44] Walsh NP. Recommendations to maintain immune health in athletes. Eur J Sport Sci 2018;18(6): 820–831. 10.1080/17461391.2018.1449895 29637836

[45] Walsh NP, Oliver SJ. Exercise, immune function and respiratory infection: An update on the influence of training and environmental stress. Immunol Cell Biol 2016;94(2): 132–139. 10.1038/icb.2015.99 26563736

[46] Brown FF, Bigley AB, Sherry C, Neal CM, Witard OC, Simpson RJ, et al Training status and sex influence on senescent T-lymphocyte redistribution in response to acute maximal exercise. Brain Behav Immun 2014;39:152–159. 10.1016/j.bbi.2013.10.031 24200513

[47] Kakanis MW, Peake J, Brenu EW, et al T helper cell cytokine profiles after endurance exercise. J Interferon Cytokine Res 2014;34(9): 699–706. 10.1089/jir.2013.0031 24673178

[48] Nielsen HG, Øktedalen O, Opstad PK, Lyberg T. Plasma cytokine profiles in long-term strenuous exercise. J Sports Med (Hindawi Publ Corp) 2016;2016:7186137 10.1155/2016/7186137 27239554 PMC4864530

[49] Pedersen BK. Special feature for the Olympics: effects of exercise on the immune system: exercise and cytokines. Immunol Cell Biol 2000;78 (5): 532–535. 10.1111/j.1440-1711.2000.t01-11-.x 11050536

[50] Pedersen BK, Toft AD. Effects of exercise on lymphocytes and cytokines. Br J Sports Med 2000;34(4): 246–251. 10.1136/bjsm.34.4.246 10953894 PMC1724218

[51] Arnold R, Brenner D, Becker M, Frey CR, Krammer PH. How T lymphocytes switch between life and death. Eur J Immunol 2006;36(7): 1654–1658. 10.1002/eji.200636197 16791883

[52] Benczik M, Gaffen SL The interleukin (IL)-2 family cytokines: survival and proliferation signaling pathways in T lymphocytes. Immunol Invest 2004;33(2): 109–142. 10.1081/imm-120030732 15195693

[53] Raeber ME, Zurbuchen Y, Impellizzieri D, Boyman O. The role of cytokines in T-cell memory in health and disease. Immunol Rev 2018;283(1): 176–193. 10.1111/imr.12644 29664568

[54] Baumann S, Dostert A, Novac N, Bauer A, Schmid W, Fas SC, et al Glucocorticoids inhibit activation-induced cell death (AICD) via direct DNA-dependent repression of the CD95 ligand gene by a glucocorticoid receptor dimer. Blood 2005;106 (2): 617–625. 10.1182/blood-2004-11-4390 15802531

[55] Schuh K, Twardzik T, Kneitz B, Heyer J, Schimpl A, Serfling E. The interleukin 2 receptor alpha chain/CD25 promoter is a target for nuclear factor of activated T cells. J Exp Med 1998;188(7): 1369–1373. 10.1084/jem.188.7.1369 9763616 PMC2212486

[56] Naseem S, Manzoor S, Javed A, Abbas S. Interleukin-6 Rescues Lymphocyte from Apoptosis and Exhaustion Induced by Chronic Hepatitis C Virus Infection. Viral Immunol 2018;31(9): 624–631. 10.1089/vim.2018.0045 30222516

[57] Moldoveanu AI, Shephard RJ, Shek PN. Exercise elevates plasma levels but not gene expression of IL-1beta, IL-6, and TNF-alpha in blood mononuclear cells. J Appl Physiol (1985) 2000;89(4): 1499–1504. 10.1152/jappl.2000.89.4.1499 11007588

[58] Smith JA, Telford RD, Baker MS, Hapel AJ, Weidemann MJ. Cytokine immunoreactivity in plasma does not change after moderate endurance exercise. J Appl Physiol (1985) 1992;73(4): 1396–1401. 10.1152/jappl.1992.73.4.1396 1447084

[59] Ullum H, Haahr PM, Diamant M, Palmø J, Halkjaer-Kristensen J, Pedersen BK. Bicycle exercise enhances plasma IL-6 but does not change IL-1 alpha, IL-1 beta, IL-6, or TNF-alpha pre-mRNA in BMNC. J Appl Physiol (1985) 1994;77(1): 93–97. 10.1152/jappl.1994.77.1.93 7961280

[60] Peake JM, Della Gatta P, Suzuki K, Nieman DC. Cytokine expression and secretion by skeletal muscle cells: regulatory mechanisms and exercise effects. Exerc Immunol Rev 2015;21: 8–25. 25826432

[61] Suzuki K, Yamada M, Kurakake S, Okamura N, Yamaya K, Liu Q, et al Circulating cytokines and hormones with immunosuppressive but neutrophil-priming potentials rise after endurance exercise in humans. Eur J Appl Physiol 2000;81(4): 281–287. 10.1007/s004210050044 10664086

[62] Bote ME, Garcia JJ, Hinchado MD, Ortega E. Fibromyalgia: anti-inflammatory and stress responses after acute moderate exercise. PLoS One 2013; 8:e74524 10.1371/journal.pone.0074524 24023948 PMC3762808

[63] Ernberg M, Christidis N, Ghafouri B, Bileviciute-Ljungar I, Löfgren M, Larsson A, et al Effects of 15 weeks of resistance exercise on pro-inflammatory cytokine levels in the vastus lateralis muscle of patients with fibromyalgia. Arthritis Res Ther 2016;18(1): 137 10.1186/s13075-016-1041-y 27296860 PMC4906587

[64] Tanghetti EA. The role of inflammation in the pathology of acne. J Clin Aesthet Dermatol 2013;6(9): 27–35. 24062871 PMC3780801

[65] Yoshimura T, Matsushima K, Tanaka S, Robinson EA, Appella E, Oppenheim JJ, et al Purification of a human monocyte-derived neutrophil chemotactic factor that has peptide sequence similarity to other host defense cytokines. Proc Natl Acad Sci U S A 1987;84(24): 9233–9237. 10.1073/pnas.84.24.9233 3480540 PMC299727

[66] Waugh DJ, Wilson C. The interleukin-8 pathway in cancer. Clin Cancer Res 2008;14(21): 6735–6741. 10.1158/1078-0432.CCR-07-4843 18980965

[67] Xie K. Interleukin-8 and human cancer biology. Cytokine Growth Factor Rev 2001;12(4): 375–391. 10.1016/s1359-6101(01)00016-8 11544106

[68] Ghafourian M, Ashtary-Larky D, Chinipardaz R, Eskandary N, Mehavaran M. Inflammatory biomarkers’ response to two different intensities of a single bout exercise among soccer players. Iran Red Crescent Med J 2016;18:e21498 10.5812/ircmj.21498 27175304 PMC4862257

[69] Petersen AM, Pedersen BK. The anti-inflammatory effect of exercise. J Appl Physiol (1985) 2005;98(4): 1154–1162. 10.1152/japplphysiol.00164.2004 15772055

[70] Fatouros IG, Jamurtas AZ. Insights into the molecular etiology of exercise-induced inflammation: opportunities for optimizing performance. J Inflamm Res 2016;9: 175–186. 10.2147/JIR.S114635 27799809 PMC5085309

[71] Malm C. Exercise immunology: a skeletal muscle perspective. Exerc Immunol Rev 2002;8: 116–167. 12690940

[72] Suzuki K, Nakaji S, Yamada M, Totsuka M, Sato K, Sugawara K. Systemic inflammatory response to exhaustive exercise. Cytokine kinetics. Exerc Immunol Rev 2002;8: 6–48. 12690937

[73] Suzuki K, Nakaji S, Yamada M, Liu Q, Kurakake S, Okamura N, et al Impact of a competitive marathon race on systemic cytokine and neutrophil responses. Med Sci Sports Exerc 2003;35(2): 348–355. 10.1249/01.MSS.0000048861.57899.04 12569227

[74] Abedelmalek S, Souissi N, Takayuki A, Hadouk S, Tabka Z. Effect of acute maximal exercise on circulating levels of interleukin-12 during ramadan fasting. Asian J Sports Med 2011;2(3): 154–160. 10.5812/asjsm.34751 22375234 PMC3289209

[75] Akimoto T, Akama T, Tatsuno M, Saito M, Kono I. Effect of brief maximal exercise on circulating levels of interleukin-12. Eur J Appl Physiol 2000;81(6): 510–512. 10.1007/s004210050076 10774876

[76] Hirose L, Nosaka K, Newton M, Laveder A, Kano M, Peake J, et al Changes in inflammatory mediators following eccentric exercise of the elbow flexors. Exerc Immunol Rev 2004;10: 75–90. 15633588

